# Sex plays a role in the construction of epiphytic bacterial communities on the algal bodies and receptacles of *Sargassum thunbergii*

**DOI:** 10.3389/fmicb.2022.935222

**Published:** 2022-07-26

**Authors:** Jing Wang, Yang Li, Zhibo Yang, Tao Sun, Xinlong Yu, Yayun Zhao, Xuexi Tang, Hui Xiao

**Affiliations:** ^1^College of Marine Life Sciences, Ocean University of China, Qingdao, China; ^2^Laboratory for Marine Ecology and Environmental Science, Qingdao National Laboratory for Marine Science and Technology, Qingdao, China

**Keywords:** sex, epiphytic bacterial community, male and female, *Sargassum thunbergii*, receptacles

## Abstract

The community structures of epiphytic bacteria on the surface of macroalgae are closely related to their host algae, but there is a lack of research on the differences between the epiphytic bacterial communities of male and female algae and their reproductive tissues. In this study, high-throughput sequencing was used to compare epiphytic bacterial community structures on the intertidal macroalgae *Sargassum thunbergii* and their receptacles between male and female samples. The epiphytic bacteria on the male and female algal bodies and receptacles had similar community structures with a large number of shared bacteria, but the samples clearly clustered separately, and the abundances of dominant taxa, specific bacteria, and indicator species differed, indicating that epiphytic bacterial communities differed significantly between the male and female *S. thunbergii* and their receptacles. In addition, the abundance of many predicted functional genes was significantly different between epiphytic bacteria on male and female algal bodies and receptacles, especially metabolism-related genes, and the abundances of predicted functional genes of epiphytic bacteria were significantly higher on both types of male samples than on female samples. Our study confirmed that the sex of the host algae influenced the epiphytic bacterial community structures on algae and algal reproductive tissues, and this role was mainly related to the host metabolism. The results reveal the role of host plant sex in the formation of epiphytic bacterial communities. These findings are helpful for obtaining an in-depth understanding of the construction mechanism of algae-associated bacteria.

## Introduction

Epiphytic bacteria living on the surface of macroalgae have formed certain community structures during long-term interaction and co-evolution with their hosts. Recognizing the composition and construction mechanism of epiphytic bacterial communities on macroalgae is a prerequisite for elucidating the complex and diverse relationships between epiphytic bacteria and host algae.

The recent studies have shown that host plants are a key driver in the construction of microbial communities (Xiong et al., 2021). Studies on algal epiphytic bacteria have also confirmed this point. Epiphytic bacterial communities are host-specific, with compositions and structures that differ significantly from those in the surrounding environment (Mancuso et al., 2016). Several studies have shown that different host macroalgae are associated with different epiphytic bacterial communities (Florez et al., 2017). This difference can be explained by the special polysaccharides secreted by the cell wall (Popper et al., 2011) and the corresponding successful colonization on algal surfaces by bacteria that can degrade and utilize such polysaccharides (Martin et al., 2014), as well as the production of secondary metabolites by different macroalgae that selectively attract or repel certain bacteria (Collén and Davison, 2001). Due to the differences in the metabolites involved in the growth and development of host algae in different taxa, microorganisms of different groups gather and form distinctive bacterial communities. Therefore, the bacterial communities of the same macroalgal species in different living environments are more similar than those of macroalgae of different species inhabiting the same niche. Hengst et al. (2010) showed that the most important factor in the community composition of epiphytic bacteria was the algal species. Therefore, the characteristics of the algal species are crucial in determining the structure of the associated bacterial community. However, even for host algae of the same species, the epiphytic bacterial community is not stable. For example, the abundances of the phyla and genera of epiphytic bacteria in *Laminaria* were found to vary greatly at different life history stages, and there were significant differences in alginic acid bacteria and cyanobacteria between the reproductive stage and the sporophyte stage (Tang et al., 2020). However, few studies have conducted comparative analyses of male and female algal epiphytic bacteria.

Dioecious plants are found in several important angiosperm taxa, and plants of different sexes not only perform different reproductive functions but also exhibit many differences in morphology, growth, and resistance to stress (Tang, 2020). In terms of morphology, there are obvious differences in plant size and reproductive organs, even in other organs. For example, the anatomical structure and epidermal morphology differ between the leaves of male and female *Podocarpus macrophyllus* (Yang et al., 2021). Physiological and biochemical indicators during development also differ between male and female plants, including biomass, net photosynthetic rate (Zhai and Sun, 2015), polysaccharide and protein (Zhang et al., 2014), flavonoids and lactones, endogenous hormone content (Ge et al., 2021), and defense enzymes (Sun et al., 2007). Differences in stress resistance are more pronounced between male and female plant individuals, with male and female plants differing in photosynthetic characteristics, antioxidant systems, and osmoregulatory capacity in response to environmental stresses (Yuan, 2018). Studies on morphological differences between male and female dioecious macroalgae have also been conducted in marine macroalgae, including *S. thunbergii* (Sun et al., 2007; Yuan, 2018), *Sargassum horneri* (Pang et al., 2018), *Sargassum fusiforme* Setch (Lin et al., 2021), and *Porphyra* (Xu, 2010). In addition, studies on the physiological and biochemical differences between male and female algae have been reported, such as the difference of the oxygen release rates between the male and female gametophytes of *Macrocystis pyrifera* (Li et al., 2005) and the difference of bromophenol concentrations between male and female reproductive tissues of *Neorhodomela larix* (Carlson et al., 1989). In addition, the content of reactive oxygen species (O^2–^ and H_2_O_2_) differs in male *S. thunbergii* and female *S. thunbergii* under enhanced UV-B radiation (Lu et al., 2022). The findings of these studies suggest that in the sexual reproduction stage of life history, dioecious algae of different sexes perform different reproductive functions through different reproductive tissues, and the physiological and biochemical differences of the algal surface microenvironment lead to the corresponding variation in epiphytic bacterial communities. Bacterial communities of interleaf microorganisms of the higher plant *Populus cathayana* have been found to be influenced by sex (Liu et al., 2021), with significant differences in the relative abundance at the genus level. Similarly, epiphytic bacterial communities of *Porphyra haitanensis* were also found to be influenced by the sex of the host plant (Yang et al., 2022). However, there have been few studies related to dioecious macroalgae. This work aims to analyze the differences in the epiphytic bacterial communities between male and female algae and their reproductive tissues and thus speculate as to whether sex has an impact on the construction of algal epiphytic bacterial communities.

*Sargassum thunbergii*, a common dominant species in the intertidal zone of northern China, is dioecious with significant differences in the morphology and internal structure of male and female receptacles during the reproductive period. The male receptacle is long and thin, with a smooth surface and white filamentous hairs on the surface of the conceptacles, whereas the female receptacle is short and thick, with a rough surface and compactly arranged cells within the tip cross-section (Yuan, 2018). *S. thunbergii* is widely used in bioremediation, aquaculture, food production, and medicine, and it has attracted increasing public attention (Wu et al., 2010). This study compared the differences between the epiphytic bacterial communities of male and female macroalgae and reproductive tissues and provided an experimental basis for clarifying the role of sex in shaping the epiphytic bacterial community of dioecious macroalgae. The findings will be beneficial for achieving an in-depth and accurate understanding of the bacteria–algae relationship.

## Materials and methods

### Sample collection and processing

The life cycle of *Sargassum thunbergii* can be divided into four periods: inactivity period, growth period, reproduction period, and senescence period (Cui, 2009). The *S. thunbergii* population of Qingdao entered a maturation period from early June to late August (Pan et al., 2011). In July 2021, *S. thunbergii* in the reproduction stage were sampled from the rocky intertidal zone of Taipingjiao (36°14′58.3′′N, 120°21′34.2′′E) in Qingdao, China, were placed into sterile sample bags, and transported to the laboratory for treatment within 30 min. *S. thunbergii* grows on large reefs in the intertidal zone of Qingdao, and the sampling area was about 5 m × 100 m. A total of 8 male and 8 female algae of healthy *S. thunbergii* were collected randomly, and each alga is a sample. At the same time, another 8 male and 8 female algae were collected, respectively, for sampling receptacles. One receptacle sample came from one individual *S. thunbergii*. The characteristics of the receptacles of *S. thunbergii* were distinguished under a microscope (Nikon H600L, Tokyo, Japan) to distinguish between male and female *S. thunbergii* (Yuan, 2018). Epiphytic bacteria sampling was conducted following a previously described method (Mathai et al., 2018). A 25 g sample of male or female *S. thunbergii* (approximately the weight of one alga), or a 25 g sample of male or female receptacles (taken from one alga using sterile forceps), was weighed, transferred to a 250-ml sterile Erlenmeyer flask, added to 70 ml of 0.01 M sterile phosphate buffer (pH 7.4), sealed with a sterile membrane, and shaken (200 r min^–1^) for 30 min at room temperature (25°C) on a shaker, after which a suspension of epiphytic bacteria was obtained. The bacterial suspension was filtered through a sterile 500-mesh sieve to remove impurities such as sediments mixed in the suspension. Then, the epiphytic bacteria were collected by vacuum filtration in a sterile environment onto 0.22-μm filter membranes and stored at −80°C. Male, female, male receptacle, and female receptacle samples were named Male-EPIP, Female-EPIP, M-EPIP-Receptacles, and F-EPIP-Receptacles, respectively, and each group had eight replicates.

### High-throughput sequencing and sequence processing

The genomic DNA extracted from the samples and the V3 + V4 regions of the 16S rDNA were amplified using specific primers with barcodes. The primer sequences were 341F: CCTACGGGNGGCWGCAG and 806R: GGACTACHVGGGTATCTAAT. Then, the polymerase chain reaction (PCR) amplification products were recovered by gel cutting and quantified using a Quantus Fluorometer (Promega, United States). The purified amplification products were mixed in equal amounts, the sequencing junctions were connected, and the sequencing library was constructed; high-throughput sequencing was performed on an Illumina HiSeq 2500 by Gene Denovo Biotechnology Co., Ltd. (Guangzhou, China).

### Data analysis

Sequences were spliced and de-duplicated using UPARSE software (v9.2.64_i86 linux32), and sequences with >97% similarity were clustered into one taxonomic operational unit (OTU). The resulting OTUs were classified using the Greengenes (gg_13.5) and SILVA (version 132) databases. The α-diversity indexes (Ace, Chao1, Shannon, and Simpson) were calculated using QIIME (v1.9.1.), and contrast analyses were carried out with Kruskal–Wallis test and Welch’s *t*-test. Meanwhile, Tukey’s honestly significant difference (HSD) was used for pairwise comparisons. A *p*-value < 0.05 was considered to have significance, and principal coordinates analysis (PCoA) and unweighted pair group method with arithmetic mean (UPGMA) were performed using the Bray-Curtis dissimilarity matrices, which were applied to analyze the β-diversity using the Vegan package in R (v2.5.3). Group differences were tested using unweighted Unifrac. Bacterial diversity was clustered at the phylum and genus levels according to the OTU classification, and species with no clear classification and species with relative abundances below 1% were recorded as other and plotted in a histogram. The specific bacterial taxa were analyzed based on the Venn diagrams obtained in R (v2.5.3) package Vegan. The software tools of linear discriminant analysis effect size (LEfSe) (v1.0) were used to identify the biomarkers with significant differences in each group. The Kruskal–Wallis rank test and the Wilcoxon rank test were used to analyze the differences between groups, and linear discriminant analysis (LDA) was used to obtain an LDA difference analysis plot, followed by an evolutionary branching plot created by mapping the differences to a classification tree with known hierarchical structure. PICRUSt (version 2.1.4) was used to predict microbial function, and Kruskal–Wallis rank-sum test (KW) was used to perform the significance analysis of functional differences.

## Results

### Biodiversity of epiphytic bacterial communities on algal bodies and receptacles of male and female *S. thunbergii*

#### α-biodiversity

A total of 32,78,089 optimized sequences were obtained after quality filtration, and the coverage of all samples was more than 99% (Supplementary Figure 1), indicating that the sequencing depth covered most species in the samples, and the sequencing data were sufficient and reliable for further analysis.

**FIGURE 1:**
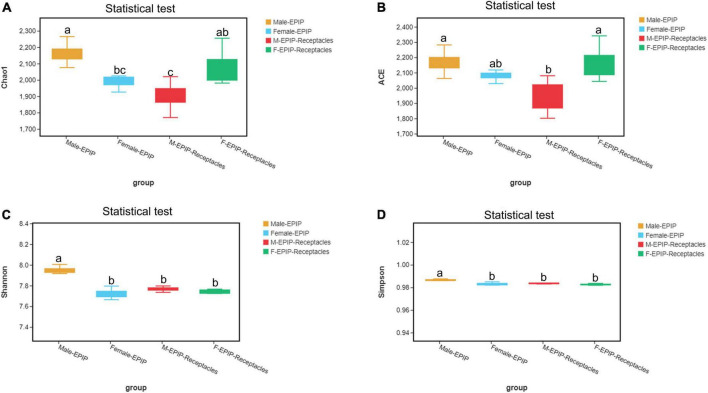
Alpha diversity of epiphytic bacterial communities on male and female *S. thunbergii* and their receptacles were assessed using the Chao1 **(A)**, ACE **(B)**, Shannon **(C)**, and Simpson **(D)** index. Different letters denote significant differences (Tukey HSD, *p* < 0.05).

The results of α-diversity analysis (Table 1 and Figure 1) showed that there were extremely significant differences between male and female samples of *S. thunbergii* and their receptacles (*p* < 0.01, by Kruskal–Wallis test). All four indexes of epiphytic bacteria on male *S. thunbergii* were significantly higher than those of other samples, indicating that the richness and diversity of bacterial community on male *S. thunbergii* were the highest (*p* < 0.05, by Welch’s *t*-test) (Supplementary Table 1). In the other three groups of samples, the Chao1 index and the ACE index were the highest on female receptacles but were the lowest on male receptacles, indicating that the richness of epiphytic bacteria on male receptacles was the lowest. However, the Shannon index and the Simpson index showed that there were no significant differences in the diversity of epiphytic bacterial community on samples from female *S. thunbergii* and female receptacles (Supplementary Table 1).

**TABLE 1:** Diversity indices of epiphytic bacterial communities on male and female *S. thunbergii* and their receptacles (Kruskal–Wallis test).

Group	Male-EPIP	Female-EPIP	M-EPIP-Receptacles	F-EPIP-Receptacles	*P*-value	Significant
Chao1	2138.37	2009.01	1900.55	2083.42	0.0006	**
Ace	2192.23	2082.85	1959.12	2171.48	0.0010	**
Shannon	7.93	7.72	7.77	7.76	0.0002	**
Simpson	0.99	0.98	0.98	0.98	0.0003	**

Asterisks (**) indicate extremely significant (p < 0.01).

#### β-diversity

**FIGURE 2:**
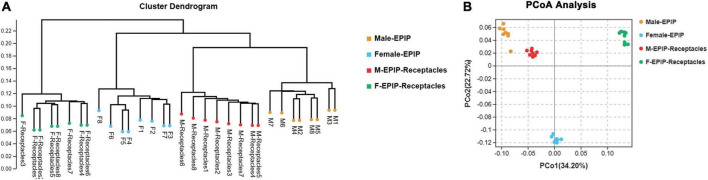
Beta diversity of epiphytic bacterial communities on algal bodies and receptacles of male and female *S. thunbergii*. **(A)** Results of the unweighted pair-group method with arithmetic mean (UPGMA). **(B)** Analysis results of principal coordinates analysis (PCoA).

The unweighted pair-group method with arithmetic mean (UPGMA) analysis and PCoA based on Bray-Curtis (Figure 2) showed that the epiphytic bacterial communities of male and female *S. thunbergii* and their receptacles could be clustered separately. The samples in each group were similar, and the difference between groups was significant (*p* < 0.01) (Table 2), indicating that there were significant differences between epiphytic bacterial communities on male and female *S. thunbergii* and their receptacles. The results of PCoA further showed that the epiphytic bacterial community on male algae was highly similar to that on the male receptacles and the least similar to that on the female receptacles (Figure 2B).

**TABLE 2:** Results of the between-group difference test at the operational taxonomic unit (OTU) level [analysis of similarities (ANOSIM) test based on unweighted Unifrac].

Diffs	*R*-value	*P*-value	Significant
Male-EPIP-vs.-Female-EPIP	0.7941	0.001	**
M-EPIP-Receptacles-vs.-F-EPIP- Receptacles	0.5458	0.001	**
Male-EPIP-vs.-M-EPIP-Receptacles	0.8571	0.002	**
Female-EPIP-vs.-F-EPIP-Receptacles	0.8097	0.001	**
Male-EPIP-vs.-Female-EPIP-vs.-M-EPIP-Receptacles-vs.-F-EPIP-Receptacles	0.7734	0.001	**

Asterisks (**) indicate extremely significant (p < 0.01).

### Composition of epiphytic bacterial communities on male and female *S. thunbergii* and their receptacles

#### Core microbiome of epiphytic bacteria and specific bacteria

**FIGURE 3:**
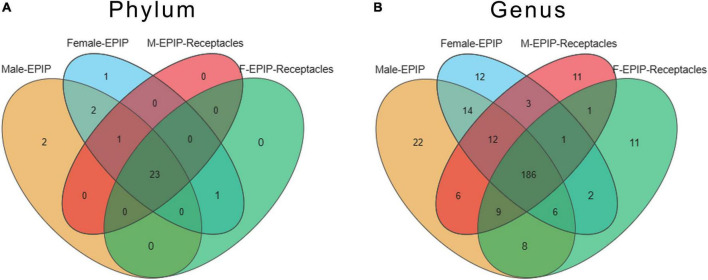
Venn diagram of epiphytic bacteria on algal bodies and receptacles of male and female *S. thunbergii* at the phylum and genus levels. **(A)** Phylum level. **(B)** Genus level.

A total of 39 phyla and 574 genera of the epiphytic bacteria were detected through high-throughput sequencing. As can be seen from the Venn diagram, there were 23 common phyla between samples from male and female *S. thunbergii* and their receptacles (Figure 3A), with the core bacterial phyla, including Bacteroidetes, Proteobacteria, and Actinobacteria (relative abundance >15%). At the genus level (Figure 3B), 186 genera were shared between all sample groups. The core genera (relative abundance >1%) were *Sva0996_marine_group* (7.56–10.00%), *Pseudoruegeria* (4.50–5.00%), *Maribacter* (2.97–5.30%), *Granulosicoccus* (2.91–3.73%), *Blastopirellula* (2.02–2.70%), and *Loktanella* (1.35–1.79%).

Specific taxa were defined as those taxa which were absent in all other groups. These specific taxa were either present in all 8 parallels or only in some of the parallels of the group. The epiphytic bacteria on male and female *S. thunbergii* each had their own specific phylum and genus, and the former had two specific phyla, Fibrobacteres and Nitrospinae, whereas the latter had only one specific phylum, Elusimicrobia. However, there was no specific phylum for samples from male and female receptacles. At the genus level, there were 22 genera specific to samples from male *S. thunbergii*, including *Methylophaga*, *Succiniclasticum*, *IS-44*, and *Prevotella_1*, whereas there were 12 genera specific to female *S. thunbergii*, including *Mycobacterium*, *Nocardioides*, and *Sulfuricurvum*. Additionally, both the samples from male and female receptacles had 11 specific genera; the samples from the male receptacles included *Planococcus*, *Weissella*, and *Actinomyces*, whereas the samples from the female receptacles included *Ochrobactrum*, *Aerococcus*, and *Frateuria* (Figure 3B). However, the proportions of these specific genera were very small. The relative abundances of *Methylophaga, Mycobacterium*, *Planococcus*, and *Ochrobactrum*, which had the highest abundances on the male algal bodies, female algal bodies, male receptacles, and female receptacles, were only 0.022, 0.006, 0.011, and 0.012, respectively.

#### Community composition and dominant epiphytic bacteria

**FIGURE 4:**
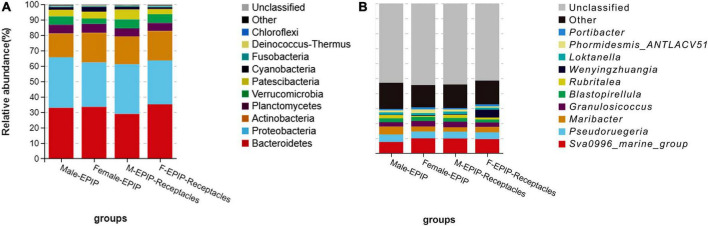
Relative abundance of epiphytic bacteria on algal bodies and receptacles of male and female *S. thunbergii.* **(A)** Phylum level. **(B)** Genus level.

The relative abundances of epiphytic bacteria on the algal bodies and receptacles of male and female *S. thunbergii* are shown in Figure 4. The results indicated that the bacterial community composition was similar between the samples from male and female *S. thunbergii* and their receptacles, but the abundances of some taxa differed significantly. At the phylum level, the dominant phyla were the same for the samples from male and female *S. thunbergii* and female receptacles, in the order of Bacteroidetes, Proteobacteria, and Actinobacteria from the most abundant to the second and third most abundant. While the dominant phyla for the samples from male receptacles were the same, the order changed to Proteobacteria, Bacteroidetes, and Actinobacteria (Figure 4A and Table 3). At the genus level, the first dominant genus in all samples was *Sva0996_marine_group*, with slightly different abundances; but the second to fourth most dominant genera were *Maribacter*, *Pseudoruegeria*, and *Granulosicoccus* in samples from both the male and female *S. thunbergii* and male receptacles, whereas the second to fourth dominant genera in the female receptacles were *Wenyingzhuangia*, *Pseudoruegeria*, and *Maribacter* (Figure 4B and Table 4).

**TABLE 3:** The top 10 of relative abundances of epiphytic bacteria on algal bodies and receptacles of male and female *S. thunbergii* at the phylum level (%).

Phylum	Male-EPIP	Female-EPIP	M-EPIP-Receptacles	F-EPIP-Receptacles
Bacteroidetes	32.88	33.47	28.90	35.15
Proteobacteria	32.75	28.84	32.25	28.44
Actinobacteria	15.45	19.17	18.00	19.06
Planctomycetes	5.61	5.78	5.29	5.21
Verrucomicrobia	5.50	3.56	5.77	5.70
Patescibacteria	4.28	4.42	6.36	3.32
Cyanobacteria	1.72	3.08	1.81	1.23
Fusobacteria	0.47	0.42	0.50	0.60
Deinococcus-Thermus	0.34	0.48	0.29	0.28
Chloroflexi	0.15	0.13	0.13	0.20

**TABLE 4:** The top 10 of relative abundances of epiphytic bacteria on algal bodies and receptacles of male and female *S. thunbergii* at the genus level (%).

Genus	Male-EPIP	Female-EPIP	M-EPIP-Receptacles	F-EPIP-Receptacles
*Sva0996_marine_group*	7.56	10.00	9.89	9.50
*Maribacter*	5.30	3.36	2.97	3.66
*Pseudoruegeria*	5.00	4.52	4.54	4.50
*Granulosicoccus*	2.91	3.73	3.71	2.93
*Blastopirellula*	2.58	2.70	2.48	2.02
*Rubritalea*	2.20	0.68	2.14	1.25
*Loktanella*	1.62	1.42	1.79	1.35
*Phormidesmis_ ANTLACV51*	1.35	2.46	1.37	0.89
*Portibacter*	0.86	1.38	0.87	1.34
*Wenyingzhuangia*	0.19	0.45	0.37	5.24

### Indicator species

**FIGURE 5:**
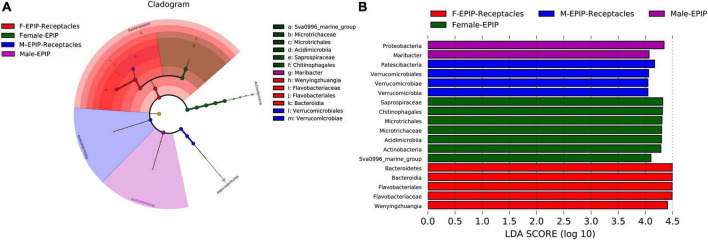
Indicator species analysis of algal bodies and receptacles of male and female *S. thunbergii*. The cladogram shows the phylogenetic structures of the microbiota. In the branching diagram of their evolution, the circles that radiate from inside to outside represent taxonomic levels from phylum to genus, and each small circle represents an individual taxon. The diameter of the circles is proportional to the relative abundance. The linear discriminant analysis (LDA) scores indicate significant differences in bacterial taxa (LDA score >4.0). **(A)** Cladogram. **(B)** LDA score chart.

Indicator species are bacterial taxa with significant differences in abundance between groups based on the analysis of linear discriminant analysis effect size (LEfSe). Indicator species analysis can reveal taxa significantly contributed to the differences between samples (Figure 5). The analysis of the LDA score (LDA > 4.0) (Figure 5A) showed that the number of indicator species in female samples was greater than that in male samples, and the indicator species of samples from algal bodies of male *S. thunbergii* were the least, consisting of only Proteobacteria (phylum) and *Maribacter* (genus), whereas those of female *S. thunbergii* were the most, including seven taxa such as Saprospiraceae (family), Chitinophagales (order), and Microtrichales (order). In addition, the indicator species of male and female receptacles consisted of four taxa, including Patescibacteria (phylum), Verrucomicrobiales (order), Verrucomicrobia (phylum), and five taxa, including Bacteroidetes (phylum), Bacteroidia (class), and Flavobacteriales (order), respectively.

**FIGURE 6:**
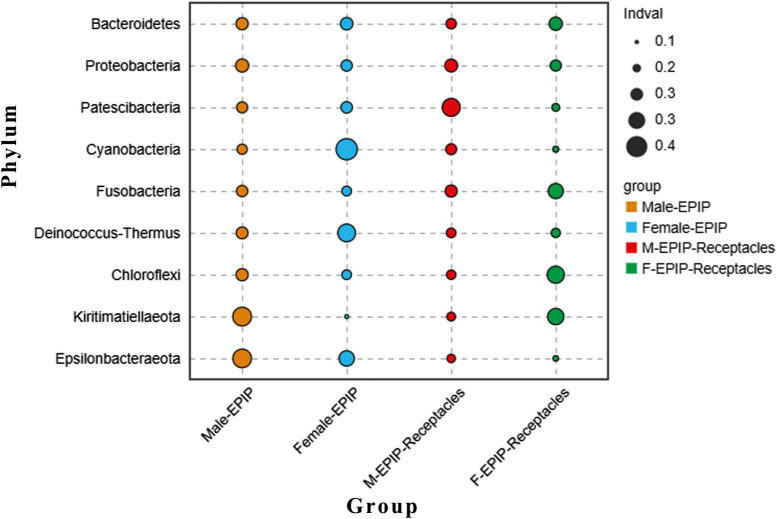
Indicator analysis of epiphytic bacteria of algal bodies and receptacles of male and female *S. thunbergii* at the phylum level.

In addition, the abundance differences of bacteria in different taxa can be analyzed by comparing their indicators. As shown in Figure 6, at the phylum level, Kiritimatiellaeota and Epsilonbacteraeota were abundant in the samples of male *S. thunbergii*, but Cyanobacteria and Deinococcus-Thermus were abundant in the samples of female *S. thunbergii*, whereas Patescibacteria were enriched in the samples from male receptacles, and Chloroflexi and Kiritimatiellaeota were enriched in the samples from female receptacles.

### Prediction of functional genes of epiphytic bacteria

**FIGURE 7:**
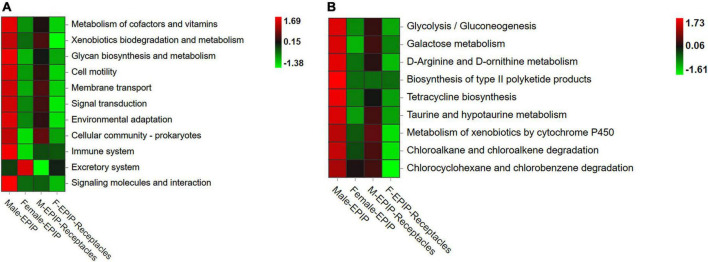
Functional prediction of partial genes of epiphytic bacteria on algal bodies and receptacles of male and female *S. thunbergii*. **(A)** Level_2 level. **(B)** Level_3 level.

The results of gene function prediction based on PICRUSt2 (Figure 7) showed that there were significant differences in predicted gene function between samples from algal bodies and their receptacles of different *S. thunbergii* sexes. At the secondary level, there were significant differences in 15 of the 34 functions analyzed by the database (*p* < 0.05, KW test). These functions mainly included metabolism (metabolism of other amino acids, xenobiotics biodegradation and metabolism, and glycan biosynthesis and metabolism), environmental information processing (signal molecule and interaction, membrane transport, and signal transduction), organismal systems (immune system, excretory system, and environmental adaptation), and cellular processes (cell motility and cell community-prokaryotes). The analysis results at the third level showed that 83 of the 171 gene functions analyzed in the database had significant differences (*p* < 0.05, KW test). The majority of these predicted genes with significant differences in abundance were related to metabolism (54 kinds), and most of these genes were related to xenobiotic biodegradation and metabolism, including 10 kinds, such as atrazine degradation and xylene degradation, followed by gene functions related to lipid metabolism with eight kinds, including secondary bile acid biosynthesis, steroid hormone biosynthesis, and fatty acid biosynthesis. Others included carbohydrate metabolism (six kinds), metabolism of cofactors and vitamins (six kinds), metabolism of other amino acids (five kinds), metabolism of terpenoids and polyketides (five kinds), biosynthesis of other secondary metabolites (four kinds), glycan biosynthesis and metabolism (three kinds), energy metabolism (three kinds), amino acid metabolism (three kinds), and nucleotide metabolism (one kind).

Almost all of the differences in the abundances of predicted genes were the highest in epiphytic bacteria on male *S. thunbergii*, followed by those on male receptacles, female *S. thunbergii*, and female receptacles (Figure 7). In contrast, the genes related to secretion were the highest in the samples from female *S. thunbergii*, followed by those of female receptacles, and were the lowest in male receptacles. In addition, the difference between male and female receptacles was not as obvious as that between male and female *S. thunbergii*.

## Discussion

### Core microbiome of epiphytic bacteria on *S. thunbergii*

In this study, it was found that the epiphytic bacterial communities differed less between samples from algal bodies and the receptacles of male and female *S. thunbergii*, and most of the bacteria in the communities were the same. Many studies have shown that the construction of epiphytic bacterial communities on the surface of algae is closely related to the host algal species (Goecke et al., 2010; Hengst et al., 2010). The core microbiomes, carrying genes with functions necessary for mutual adaptation with their hosts, are stable and consistent components of bacterial communities (Shade and Handelsman, 2012). Identifying the core microbiome of algal epiphytic bacteria is important for understanding the roles played by key microorganisms and the interactions between bacteria and host algae.

In this study, numerous epiphytic bacteria were shared between male and female *S. thunbergii* and their receptacles. At the phylum level, the core bacteria were Bacteroidetes, Proteobacteria, and Actinobacteria. This finding was similar to the results of previous studies on marine macroalgae (Florez et al., 2017). At the genus level, the six genera with abundances of more than 1% consisted of *Sva0996_marine_group*, *Pseudoruegeria*, *Maribacter*, *Granulosicoccus*, *Blastopirellula*, and *Loktanella*; these genera were completely different from the common core genera of terrestrial plants (Trivedi et al., 2020) but were similar to the core genera identified in *Laminaria digitata* (*Blastopirellula*, *Litorimonas*, and *Sva0996 marine group*) (Ihua et al., 2020). It has been reported that nutrient cycling between algae and bacteria is important for maintaining a long and stable relationship between algae and bacteria (Christie-Oleza et al., 2017). Most of the core bacteria of *S. thunbergii* are closely related to algal source materials and possess a strong metabolic capacity. For example, *Sva0996_marine_group*, with the highest abundance, is a common taxon that utilizes organic matter in the marine environment, and it has been reported to assimilate dissolved proteins derived from phytoplankton (Orsi et al., 2016; Wang et al., 2018); *Loktanella* is also an important dimethylsulfoniopropionate (DMSP) degrader (Sun et al., 2020); *Granulosicoccus* contains well-known hydrocarbon degraders (Rizzo et al., 2019); *Blastopirellula* is involved in the oxidation of ammonia and is associated with the nitrogen cycle (Tian et al., 2015); and *Pseudoruegeria* is associated with lipid (Park et al., 2018) and particulate organic carbon (POC) metabolism (Sun, 2018). In addition, these bacteria have some beneficial effects on algae; for example, *Maribacter* is the most important bacterial group associated with algal morphogenesis (Ghaderiardakani et al., 2019), and *Pseudoruegeria* has been reported to degrade polycyclic aromatic hydrocarbons, purify the surrounding environment of algae, and indirectly benefit the growth of algae (Yuan, 2008). However, not all of these core groups are beneficial to seaweeds. For example, *Maribacter*, *Loktanella* spp., and *Acinetobacter* spp. had been reported to have inhibitory effects on algae (Zheng et al., 2019).

In addition, there were also some shared bacteria that were low in abundance but important in function. These include *Phaeobacter* has been reported to have a dual action on algae, providing growth hormones to the algae at the early stage of growth of the host *Emiliania huxleyi*. When the host algae decay, the bacteria synthesize algae-lysing components (Roseobacticides) to accelerate the dissolution of the algae (Seyedsayamdost et al., 2014; Zhang et al., 2018). Therefore, it could be concluded that the relationships between the epiphytic bacterial community and the host algae *S. thunbergii* and their receptacles were very complex; not all of these relationships were mutually beneficial, and the functions of bacteria with low abundances were also very important in the community.

### Role of sex in the construction of epiphytic bacterial communities in male and female *S. thunbergii* and their receptacles

The influence of host plant sex on epiphytic bacterial communities has been verified in the higher plant *Populus cathayana* (Liu et al., 2021). Additionally, some bacteria were differentially enriched on the male and female *Porphyra haitanensis* (Yang et al., 2022). This study detected the differences in the diversity, specific taxa, and indicator species of the epiphytic bacterial communities, verifying that sex was a driving factor in the construction of the epiphytic bacterial community of *S. thunbergii* and its reproductive tissues. The results of the diversity analysis showed that the epiphytic bacterial communities could be clustered separately between algal bodies and the receptacles of male and female *S. thunbergii*. There were significant differences between samples of the different sexes of *S. thunbergii* and their receptacles, and the richness of epiphytic bacteria was the highest in the male *S. thunbergii* and the lowest in the male receptacles. PCoA showed that the epiphytic bacterial communities of male *S. thunbergii* and male receptacles differed, but the differences between communities were small, while those of female *S. thunbergii* and female receptacles differed greatly, indicating that female receptacles had stronger filtering and selection effects on epiphytic bacteria. This may be due to the physiological functions of female receptacles during sexual reproduction in *S. thunbergii* are more complex than those of male receptacles. Oviposition and fertilization are completed in female receptacles, and the fertilized eggs fall from the female receptacles only after they have developed into embryo seedlings. As a result, their surface microenvironment and metabolites are more complex than those of the male receptacles, leading to a greater difference between the epiphytic bacteria on the surface of the female receptacles and the female algae (Yuan, 2018).

In this study, most of the specific epiphytic bacteria of algal bodies and receptacles of male and female *S. thunbergii* were related to metabolism, indicating that there were differences in the metabolic capacity of *S. thunbergii* and their receptacles of different sexes. For example, at the phylum level, Elusimicrobia, which was specific to samples of female *S. thunbergii*, had nitrogen-fixing enzymes (type IV) associated with nitrogen fixation (Zheng et al., 2016). Regarding the two phyla specific to samples of male *S. thunbergii*, members of Nitrospinae have been reported to influence the carbon cycle in deep-sea regions by synthetically participating in the nitrite cycle (Pachiadaki et al., 2017), and members of the Fibrobacteres phylum can efficiently degrade cellulose (Jewell et al., 2013). No specific phyla were identified in samples from male and female receptacles, which indicated that the bacterial richness of receptacles was less than that of algae and also verified that the epiphytic bacteria would be filtered and selected from algae to reproductive tissues.

At the genus level, the specific epiphytic bacteria of each group were closely associated with algal physiological activities. The specific epiphytic bacteria of male *S. thunbergii* had the ability to degrade and synthesize a wide range of substances. *Methylophaga*, with the highest abundance, is a dimethylsulfur-degrading bacterium (Schäfer, 2007), followed by succinate degrading bacterium *Succiniclasticum* (Rainey, 2015) and *Prevotella*_1 (Ivarsson et al., 2014), which is involved in the fermentation of proteins and carbohydrates. The specific epiphytic bacteria also included the grease-degrading bacterium *Alkanindiges* (Ron and Rosenberg, 2010), the carbohydrate-degrading bacterium *Succinivibrio* (Bryant, 2015), the polysaccharide-degrading bacterium *Pseudofulvibacter* (Yoon et al., 2013), and the pollutant-degrading bacterium *Luminiphilus* (Sun et al., 2015). Bacteria that could synthesize substances were also abundant, including *Methylophaga*, which produces growth hormone (indole-3-acetic acid) (Li et al., 2007), *Limnothrix*, which produces phycocyanin (C-PC) (Gantar et al., 2012), *Owenweeksia* (Bowman, 2015), which produces carotenoids, and *Solibacillus* (Markande et al., 2013), which produces flagellin-like proteins.

Compared with male algal samples, female *S. thunbergii* had fewer specific epiphytic bacteria. Among them, *Mycobacterium* was the most abundant, being a human pathogenic bacterium (Gagneux et al., 2006) also reported to degrade pyrene (Luo, 2010), followed by *Nocardioides*, which could utilize a wide range of carbon and nitrogen sources, including organic compounds and toxic environmental pollutants (Evtushenko et al., 2015), and was also associated with antimicrobial properties, such as inhibiting plant pathogen *Ralstonia solanacearum* (Jiang, 2019). Other metabolizing bacteria included the sulfur-oxidizing bacterium *Sulfuricurvum* (Zhao, 2019), the organic matter-degrading bacterium *Thauera* (Heider and Fuchs, 2015), the protein-degrading bacterium *Aliikangiella* (Wang et al., 2020), the fatty acid metabolizing *Acuticoccus* (Zheng et al., 2021), and the pollutant-degrading bacterium *Cloacibacterium* (Hassan et al., 2018). However, the genus number of specific bacteria that synthesized substances on the female algae was much lower than that on the male algae, with only one genus—the carotenoid-synthesizing bacterium *Algoriphagus* (Nedashkovskaya and Vancanneyt, 2015).

Both male and female receptacle samples had 11 kinds of specific genera, but there were fewer epiphytic bacterial genera related to metabolism in male receptacles; these consisted of only *Planococcus* (Li et al., 2006), an aromatic hydrocarbon-degrading bacterium with the highest abundance, *Anaerococcus* (Ezaki and Ohkusu, 2015), which metabolized amino acids, proteins, and carbohydrates, and *Trichococcus*, which can metabolize polysaccharides, amino acids, and cellulose, etc. (Li M. et al., 2022). The specific genera included a variety of human pathogens such as *Enterococcus* (Murray, 1990), *Francisella* (Keim et al., 2007), and the algolytic *Gayadomonas* (Jung et al., 2017). The specific genera also included *Weissella* (Tian and Zhang, 2021), which inhibits the growth of harmful microorganisms, the antibiotic-producing *Actinomyces* (An et al., 2002), and the protease- and bacteriocin-producing *Lactococcus* (Klijn et al., 1995), suggesting that the epiphytic bacteria on male receptacles had fewer metabolic functions compared to other sample groups but had a variety of functions related to pathogenicity and antagonism.

The specific genera of epiphytic bacteria in female receptacles were very different from those in male receptacles. *Ochrobactrum* (Naik et al., 2013) and *Aerococcus* (Rasmussen, 2016), with great abundance, are human pathogens. In addition, there were some degrading bacteria, such as brown algae polysaccharide-degrading bacteria *Formosa* (Kusaykin et al., 2017), sulfate-reducing bacteria *Desulfobacter* (Kuever et al., 2015), and dissimilatory iron (III)-reducing bacteria *Deferrisoma* (Slobodkina et al., 2012). It is interesting to note that both *Ochrobactrum* (Ghosal et al., 2010) and *Frateuria* are phenol-degrading bacteria (Zemb et al., 2012). Some studies have shown that the content of phenols in female plants is significantly different from that in male plants (Li and Yang, 2012) and bromophenol concentrations were higher in female reproductive structures than in male reproductive structures of the red algae *Neorhodomela larix* (Carlson et al., 1989). These two genera of phenol-degrading bacteria were specific to the female receptacles, probably because the type and abundance of phenol released from this tissue led to a corresponding increase in the abundance of phenol-degrading bacteria in the female receptacles. In addition, *Eudoraea* (Banta et al., 2017) and *Gilvibacter* (Kim, 2006) are capable of synthesizing arborane triterpenols and proteorhodopsin, respectively, which are related to important physiological functions of bacteria. These taxa were not found in other sample groups, which was speculated to be related to the special physiological function of female receptacles.

In addition to the differences in specific bacteria, the indicator species also indicated the presence of taxa with significant differences in abundance between the sample groups. These bacteria were also closely related to algae. For example, the indicator species of male *S. thunbergii* were Proteobacteria (phylum), with complex degradation ability, and *Maribacter* (genus), which promotes morphological development and algal inhibition (Ghaderiardakani et al., 2019; Zheng et al., 2019). The indicator species with the highest indicator value of endophytic bacteria in female *S. thunbergii* was Saprospiraceae (family), which has the ability to hydrolyze and utilize complex carbon sources and can metabolize complex organic compounds (McIlroy and Nielsen, 2014; Zheng X. et al., 2018). Another indicator species, Chitinophagales (order), can degrade recalcitrant carbon (Li Y. et al., 2022). The main indicator species of male receptacles was Patescibacteria (phylum), which is related to calcium ion exchange and heavy metal degradation (Song et al., 2021). The abundance of Patescibacteria on male receptacles was about two times that on female receptacles. Patescibacteria have the same calcium ion exchange function as Omnitrophicaeota, which was absent in the samples from female receptacles, but how this function is related to the absorption and utilization of calcium by male and female receptacles remains to be further verified. Other indicator species, namely, Verrucomicrobiales (order) and Verrucomicrobia (phylum), have the ability to decompose mucin (Singh and Natraj, 2021). Among the indicator species of female receptacles, Bacteroidetes and Bacteroidia are considered to be the main degraders of algal polysaccharides and also degrade a variety of substances such as proteins, lipids, and cellulose, which are important in the biogeochemical cycling of marine substance materials (He et al., 2018), whereas Flavobacteriales (order) prefer attachment growth, easily degrade high molecular weight organic substances of algal origin, and often appear during the growth stage in which algae release high molecular weight dissolved organic carbon (DOC) and POC (Zhang et al., 2018; Zheng Q. et al., 2018). The abundance of *Wenyingzhuangia* in the female receptacles was about 15 times that in the male receptacles. *Wenyingzhuangia* has been reported to have the ability to produce agarose (Tian et al., 2019). Whether this means that the female receptacles of *S. thunbergii* release more polysaccharides and agar needs to be verified by relevant studies in combination with research on the differences in the physiological and biochemical metabolism between male and female receptacles of *S. thunbergii*.

### Functional differences of predicted genes of epiphytic bacteria in algal bodies and receptacles of male and female *S. thunbergii*

The above discussion of specific bacteria and indicator species showed that the epiphytic bacteria in algal bodies and receptacles of male and female *S. thunbergii* differed in nutrient utilization, pollutant degradation, antibacterial activity, and the synthesis of some functional substances, but the greatest differences were found in material metabolism. It can be therefore hypothesized that the differences in epiphytic bacterial communities between the different sexes of algae and receptacles are mainly related to the metabolic processes of algae and their reproductive tissues.

However, bacteria are not classified according to the physiological functions they perform. Therefore, in the previous analysis, multiple bacteria of the same taxon may perform different functions, and the same function may be performed by different bacterial taxa (Florez et al., 2017). Although our study has clearly shown that the bacterial communities of algal bodies and receptacles of different sexes are obviously clustered, respectively, indicating that sex had an effect on the epiphytic bacterial community of algae and algal reproductive tissues, it was difficult to elucidate the mechanisms by which the differences in algae and the reproductive tissues of different sexes led to different epiphytic bacteria due to the lack of relevant studies on metabolic differences between male and female host algae and their reproductive tissues. By analyzing the differences in the predicted gene abundance of epiphytic bacteria, the differences in the physiological functions of epiphytic bacteria of samples of different sexes can be inferred.

The results of this study revealed that there were significant differences in the abundances of predicted functional genes of epiphytic bacteria between different sexes of *S. thunbergii* and their receptacles, and the differences were mainly found in metabolism-related functions. At the secondary and tertiary levels, metabolism-related genes accounted for a large proportion of the significantly different genes, which also indicated that the metabolic ability of the epiphytic bacteria on algal bodies and receptacles of male and female S. *thunbergii* differed significantly. This difference might be the reason why bacteria with different metabolic abilities are able to coexist on the surface of algae and on the receptacles of a certain sex. In addition, the abundance of predicted functional genes was mostly higher in male samples than in female samples and also higher in algal samples than in receptacle samples. Numerous studies have concluded that the growth rate, environmental adaptability, and resistance of male plants exceed those of female plants (Tang, 2020). The differences in the predicted abundance of genes related to metabolic functions (including nutrient utilization and contaminant degradation) of epiphytic bacteria on *S. thunbergii* between the two sexes were consistent with these earlier findings, which indicated that the differences in metabolism between male and female host plants were an important factor in the differences in epiphytic bacterial communities on algae of different sexes. In addition, the anabolism of substances in the reproductive tissues of plants was very strong, because a large amount of substances needed to be prepared for the reproduction and division of germ cells. In contrast, the catabolism was relatively low compared with other parts of plants, resulting in the metabolic functions of epiphytic bacteria closely related to decomposition products being lower than that of algal epiphytic bacteria. This may have been the reason why the predicted gene functional abundance of epiphytic bacteria on the receptacles was lower than that of the algal epiphytic bacteria. However, because the differences between male and female algae and their receptacles have been studied less at the physiological and biochemical levels, further study should focus on how to establish a precise link between epiphytic bacterial taxa and the types and amounts of metabolized substances of male and female algae and their receptacles.

## Conclusion and future prospects

In this study, the characteristics of epiphytic bacterial communities on the algal bodies and receptacles of male and female *S. thunbergii* were analyzed. It was found that the interactions between core bacteria and host algae were complex. In addition, the findings showed that sex played a role in the construction of epiphytic bacterial communities on *S. thunbergii* and their receptacles. It is speculated that the difference in substance metabolism between male and female samples of host algae and their receptacles was the main factor leading to the differences in the epiphytic bacterial community. Future work will investigate the differences between male and female epiphytic bacterial communities on other marine macroalgal species in parallel with the physiological and biochemical indicators of the host algae with the help of metabolomics, to better elucidate the mechanisms of host algal sex affecting the community structure of epiphytic bacteria on host algae.

## Data availability statement

The datasets presented in this study can be found in online repositories. The names of the repository/repositories and accession number(s) can be found below: https://www.ncbi.nlm.nih.gov/, SAMN27611150, https://www.ncbi.nlm.nih.gov/ (SAMN27611151–SAMN27611181).

## Author contributions

JW, XT, and HX conceived and designed the experiments. JW, YL, ZY, and TS performed the experiments. JW and YL performed the statistical analyses and wrote the manuscript. JW, YL, ZY, TS, XY, YZ, XT, and HX edited the manuscript. All authors discussed the results, read, and approved the final version of the manuscript.
