# Supplementary material for: Sex plays a role in the construction of epiphytic bacterial communities on the algal bodies and receptacles of Sargassum thunbergii
Source: Front Microbiol. 2022 Jul 26;13:935222. doi: 10.3389/fmicb.2022.935222 (PMC9360977; doi:10.3389/fmicb.2022.935222)
Supplement: Supplementary file 1 [file Data_Sheet_1.docx]

Supplementary Material

# Supplementary Figures and Tables

## Supplementary Figure


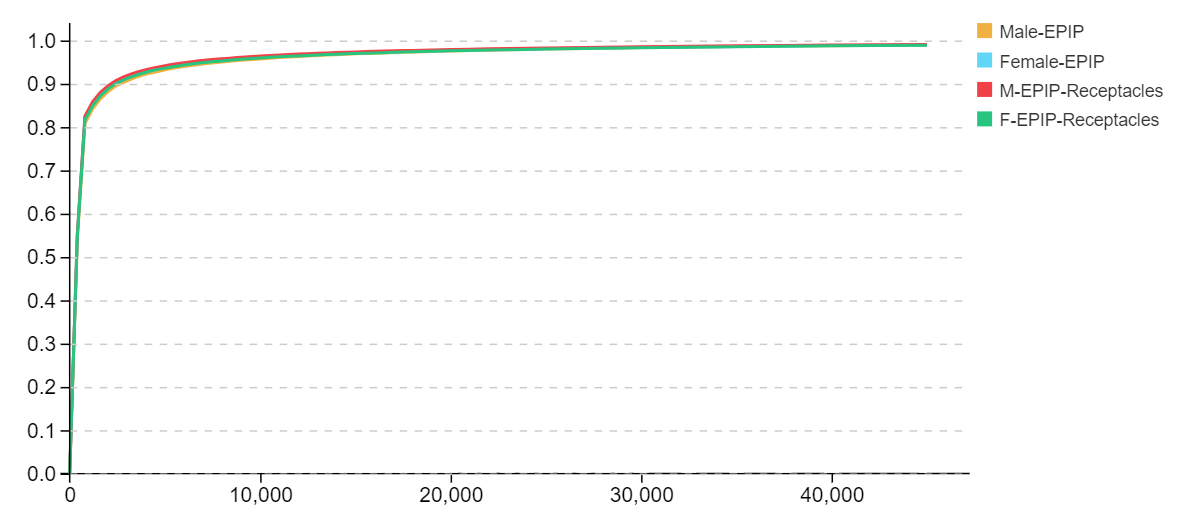


**Supplementary Figure 1.** Dilution curves of the coverage of epiphytic bacteria on male and female *Sargassum thunbergii* and their receptacles

## Supplementary Table

**Supplementary Table 1** P-value calculated using Welch’s T-test of diversity indices of epiphytic bacterial communities on male and female *Sargassum thunbergii* and their receptacles

| Group | Chao1 | ACE | Shannon | Simpson |
| --- | --- | --- | --- | --- |
| Male-EPIP-VS-Female-EPIP | 0.004365 | 0.025298 | 0.000001 | 0.000013 |
| M-EPIP-Receptacles-VS-F-EPIP-Receptacles | 0.000986 | 0.000985 | 0.569418 | 0.091519 |
| Male-EPIP-VS-M-EPIP-Receptacles | 0.000043 | 0.000285 | 0.000028 | 0.000493 |
| Female-EPIP-VS-F-EPIP-Receptacles | 0.093173 | 0.079071 | 0.124714 | 0.382522 |
